# Analysis of renal cancer cell lines from two major resources enables genomics-guided cell line selection

**DOI:** 10.1038/ncomms15165

**Published:** 2017-05-10

**Authors:** Rileen Sinha, Andrew G. Winer, Michael Chevinsky, Christopher Jakubowski, Ying-Bei Chen, Yiyu Dong, Satish K. Tickoo, Victor E. Reuter, Paul Russo, Jonathan A. Coleman, Chris Sander, James J. Hsieh, A. Ari Hakimi

**Affiliations:** 1 Department of Computational Biology, Memorial Sloan-Kettering Cancer Center, 417 E 68th St, New York, New York 10065, USA; 2 Department of Genetics and Genomic Sciences, Icahn Institute of Genomics and Multiscale Biology, Icahn School of Medicine at Mount Sinai, One Gustave L. Levy Place, Box 1498, New York, New York 10029, USA; 3 Urology Service, Department of Surgery, Memorial Sloan-Kettering Cancer Center, 417 E 68th St, New York, New York 10065, USA; 4 Department of Pathology, Memorial Sloan-Kettering Cancer Center, 417 E 68th St, New York, New York 10065, USA; 5 Human Oncology and Pathogenesis Program, Memorial Sloan-Kettering Cancer Center, 417 E 68th St, New York, New York 10065, USA; 6 cBio Center, Department of Biostatistics and Computational Biology, Dana-Farber Cancer Institute and Compbio Collaboratory, Department of Cell Biology, Harvard Medical School, 450 Brookline Avenue, Boston, Massachusetts 02215-5450, USA; 7 Molecular Oncology, Department of Medicine, Siteman Cancer Center, Washington University, St. Louis, Missouri 63110, USA

## Abstract

The utility of cancer cell lines is affected by the similarity to endogenous tumour cells. Here we compare genomic data from 65 kidney-derived cell lines from the Cancer Cell Line Encyclopedia and the COSMIC Cell Lines Project to three renal cancer subtypes from The Cancer Genome Atlas: clear cell renal cell carcinoma (ccRCC, also known as kidney renal clear cell carcinoma), papillary (pRCC, also known as kidney papillary) and chromophobe (chRCC, also known as kidney chromophobe) renal cell carcinoma. Clustering copy number alterations shows that most cell lines resemble ccRCC, a few (including some often used as models of ccRCC) resemble pRCC, and none resemble chRCC. Human ccRCC tumours clustering with cell lines display clinical and genomic features of more aggressive disease, suggesting that cell lines best represent aggressive tumours. We stratify mutations and copy number alterations for important kidney cancer genes by the consistency between databases, and classify cell lines into established gene expression-based indolent and aggressive subtypes. Our results could aid investigators in analysing appropriate renal cancer cell lines.

## Abstract (web summary)

Cell lines are central to cancer research, but knowing which cell lines are the best representative of actual tumours is a major challenge. Here the authors provide a resource assessment of 65 renal cell lines to assist researchers in selecting suitable lines for studying specific renal carcinoma subtypes.

Over the past six decades, immortalized cancer cell lines have had an increasingly important role in the study of cancer biology and response to therapeutics. Ideally, a cell line should closely resemble the particular cancer type of interest in order to serve as a suitable *in vitro* model for investigation. However, studies have identified molecular differences between commonly used cancer cell lines and human tumour samples^1^,^2^,^3^,^4^,^5^. With the maturation of various Cancer Genome Atlas (TCGA) studies, genomic characterization and expression data for more than 30 cancer types have been reported to date^6^. In addition, the Broad-Novartis Cancer Cell Line Encyclopedia (CCLE)^7^,^8^ and the COSMIC Cell Lines Project (CCLP)^8^,^9^,^10^ each provide publicly available mutation information, DNA copy number, and mRNA expression profiles for more than 1,000 cancer cell lines.

With such data now publicly accessible, efforts have been initiated to compare the genomic similarity of commonly used cell lines to known tumour samples. Previous work from our laboratory comparing data from TCGA and CCLE for high-grade serous ovarian cancer (HGSOC) revealed differences between some of the most commonly used cell lines and HGSOC tumour profiles. Additionally, we demonstrated that several cell lines initially classified or widely used as HGSOC were probably derived from other ovarian cancer subtypes^11^. A similar analysis was reported on head and neck squamous cell carcinoma cell lines^12^.

Renal cell carcinoma (RCC) is the eighth leading cause of cancer-related death in the US and has an annual incidence of more than 270,000 new cases globally^13^. RCC is subdivided into several histological subtypes with unique genomic profiles and clinical implications^14^. Ongoing efforts by the TCGA continue to identify the most common mutational aberrations for the various histological subtypes. Clear cell RCC (ccRCC) is the most common (∼80%) subtype and is characterized by bi-allelic loss of tumour suppressor genes on chromosome 3p, the most common of which are *VHL*, *PBRM1*, *SETD2* and *BAP1* (refs 15, 16). Recurrent copy number alterations (CNAs) of chromosomes 5, 8 and 14 have been identified as additional pathogenic mechanisms of ccRCC^15^,^17^,^18^. With a frequency of ∼15%, papillary RCC (pRCC) is the second most common subtype of malignant kidney tumours^19^. Activating germline and somatic mutations of the *MET* oncogene at 7q31 and amplifications of chromosomes 7 and 17 have been implicated in the oncogenesis of type I pRCC^20^,^21^,^22^. Finally, chromophobe RCC (chRCC) accounts for ∼5% of all RCCs and is typically more indolent in disease course than ccRCC and pRCC^23^. TCGA analysis has revealed that chRCC has a unique molecular pattern based on loss of one copy of the entire chromosome for most or all of chromosomes 1, 2, 6, 10, 13, and 17; however, focal copy number events were absent indicating a less complex genetic profile than other kidney cancers^24^.

Utilizing these three rich data sets (CCLE, CCLP and TCGA) we characterize commercially available RCC cell lines with respect to genomic resemblance to human RCC. We further classify the cell lines resembling ccRCC into prognostic groups based on the validated ccA and ccB expression-based subtypes^25^,^26^.

In our comparison of RCC molecular profiles from TCGA, CCLE and CCLP data, we characterize individual commercially available RCC cell lines and help to distinguish their sub-histology as well as their resemblance to human RCC. These findings may help future investigators select the most appropriate cell line tailored to the RCC subtype under examination.

## Results

### Similarity of cell lines common to CCLE and CCLP

**Figure 1:**
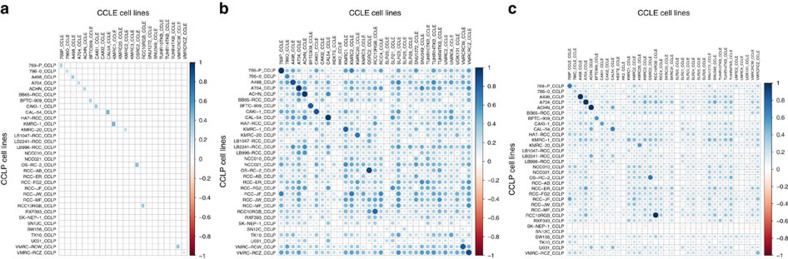
Comparison of CCLE and CCLP kidney cell lines using genomic data. (**a**) Comparison of binary mutation data using the Jaccard similarity index (**b**). Comparison of CNAs using Pearson's correlation coefficient, and (**c**). Comparison of mRNA gene expression data using Pearson's correlation coefficient. Matching cell lines show higher similarity than non-matching cell lines for each data type, and the similarity between cell lines is appreciably higher using copy number or gene expression data than it is using mutation data.

We compared the kidney cell lines from CCLE and CCLP using mutation, CNA and gene expression data (Table 1), after pre-processing to make the data comparable (see Methods). While the similarity between the 14 cell lines common to CCLE and CCLP is higher than their similarity to all other cell lines for CNA and gene expression data, the mutation data agrees to a lesser extent (Fig. 1). However, the inter-dataset similarity is nonetheless higher for common cell lines, albeit lower than that for gene expression and CNA data. This is in agreement with recent work^27^ reporting discrepancies in the detection of missense mutations in cell lines common to CCLE and CCLP (57% conformity). SK-NEP-1 was strikingly unlike the other cell lines, showing near-zero or even slight negative correlation with most other cell lines using gene expression data (Fig. 1c) – this might not be surprising as it has been reported to be an Ewing family tumour line^28^, even though CCLP only lists it as a kidney cell line of unspecified histological subtype (NS).

### Clustering by CNAs reveals distinct RCC subtypes

**Figure 2:**
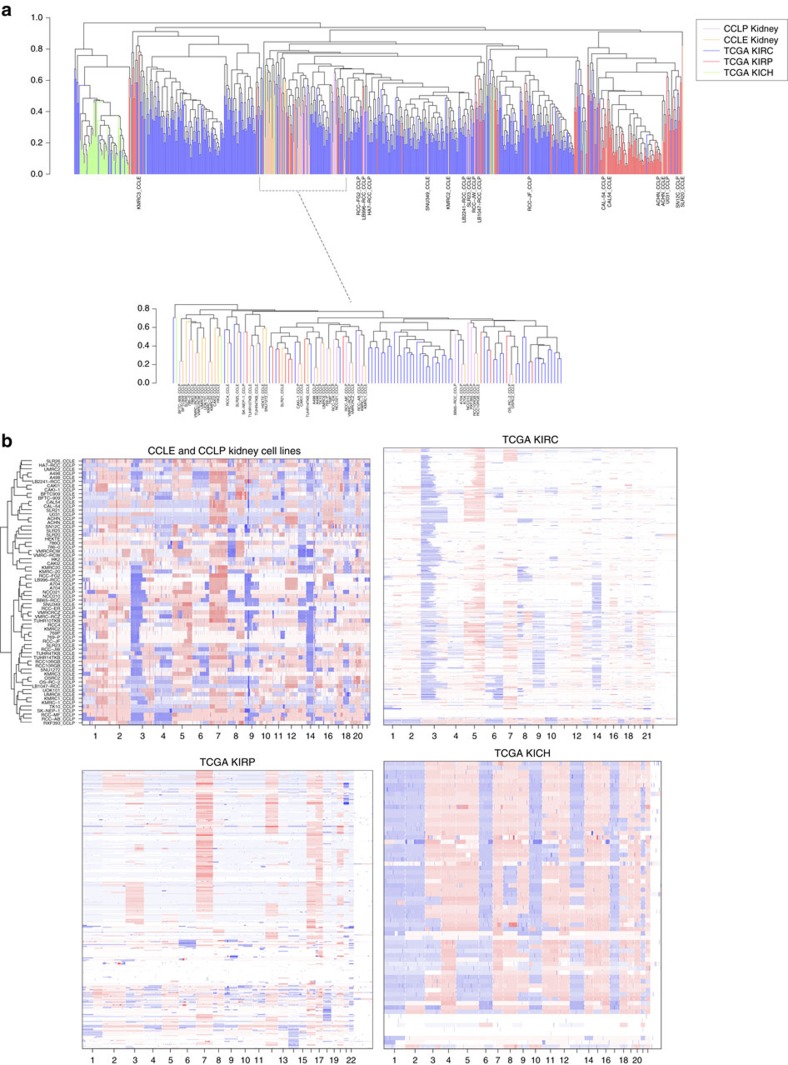
Clustering RCC cell lines and tumours by CNAs into RCC subtypes. (**a**) CNA-based clustering of 32 CCLP and 33 CCLE kidney cell lines and 728 TCGA kidney tumours (504 KIRC or clear cell, 158 KIRP or papillary and 66 KICH or chromophobe). Tumours clearly separate by subtype and the majority of cell lines cluster with clear cell renal tumours. No cell lines cluster with chromophobe tumours, but 3, ACHN, U031 and CAL54, cluster with papillary tumours. Two cell lines—SN12C and SLR21–are outliers and cluster away from all other tumours and cell lines on their own. (**b**) CNA landscape of CCLE and CCLP kidney cell lines–most of the clear cell renal cell lines show the characteristic 3p loss and *VHL* mutations (refer to Fig. 3), while several show other characteristic CNAs. ACHN, U031 and CAL54 show characteristic pRCC alterations, while SN12C and SLR21 are unlike any of the tumour subtypes. Cell lines are ordered according to the clustering in **a**, so cell lines with shared alterations are together. The CNA landscapes of the TCGA KIRC, KIRP and KICH data sets are also shown for comparison.

Due to the distinct copy number profiles of the common subtypes of RCC, we compared 33 kidney-derived cell lines from CCLE and 32 from CCLP to all 728 TCGA kidney cancer tumours (504 ccRCC, 158 pRCC and 66 chRCC) using CNA data. Our analysis reveals that the cell lines cluster according to well-described RCC subtypes (Fig. 2). After excluding the CCLE cell lines that originate from normal renal epithelium (HEKTE and HK-2), the vast majority (28/31, 90% of CCLE, and 28/32, 87.5% of CCLP) cluster with the ccRCC sub-histology. ACHN and CAL54 (from both CCLE as well as CCLP), as well as U031 from CCLP cluster with pRCC, while SN12C from CCLP and SLR20 from CCLE cluster on their own, as outliers with some similarity to pRCC. Of note, none of the available cell lines in the CCLE or CCLP cluster with the chRCC subtype.

Intriguingly, our comprehensive review of the literature in PubMed Central identified ACHN as the third most highly cited RCC cell line despite the fact that it clusters with pRCC (Fig. 2a and Supplementary Table 1). SN12C is another highly cited cell line that does not cluster with ccRCC—which might be due to it having been established from a RCC with extensive invasion of perinephric fat, and displaying a mix of clear cell and poorly differentiated RCC^29^. The remaining eight out of the top 10 most highly cited cell lines cluster with ccRCC, but it is worth noting that TK-10, while displaying 3p loss, shows a rather unusual CNA landscape, with several arm-level gains and losses that are not characteristic of ccRCC. TK-10, while often used as a ccRCC cell line, was originally reported to be from a tumour with cells of an epithelial nature, with papillary and glandular structure, as well as a spindle pattern^30^—characteristics that are suggestive of aggressive sarcomatoid RCC. Figure 2b shows CNA heatmaps for all the cell lines, along with kidney renal clear cell carcinoma (KIRC), kidney papillary (KIRP) and kidney chromophobe (KICH) tumours, illustrating shared alterations among the cell lines as well as their resemblance to the tumour CNA profiles.

### Tumours resembling cell lines bear hallmarks of aggression

It is evident that clustering analysis based on CNAs demonstrated that a subset of ccRCC tumours clusters away from the cell lines, while others cluster with cell lines. We compared these subsets in order to determine the properties of tumours that cell lines might best represent. This revealed that the tumours clustering with cell lines tend to be of higher stage (Stages 1/2: 43.6 versus 67.2%, Stages 3/4: 56.3 versus 32.8%, *P* value 0.003, Fisher's exact test), higher grade (grades G1/G2: 31.2% vs 51.9%, grades G3/G4: 68.7% vs 48.1%, *P* value 0.049, Fisher's exact test). These tumours also display a higher extent of copy number aberrations (mean fraction genome altered: 19% versus 12%), and more frequent mutations in genes such as *BAP1* (12.3% versus 5.4%), *SETD2* (12.7% versus 8.1%) and *MTOR* (6% versus 4.7%), which are associated with more aggressive disease (though only *BAP1* has a statistically significant difference—*P* value 0.025, Fisher's exact test).

These results indicate that the tumours that are likely to better represented by the cell lines display clinical and genomic features corresponding to more aggressive disease.

Since the subset of tumours that cluster with cell lines can vary depending on the parameters used in clustering, we also repeated the analysis by comparing the tumours in the top and bottom quartiles by mean correlation of CNA profiles with the cell lines, which yielded consistent results.

### Comparison of mutations between RCC cell lines and tumours

To compare copy number and mutational profiles of RCC cell lines to human tumours we used available single-nucleotide polymorphism (SNP) array and targeted exome data for 415 ccRCC tumours. As expected, our analysis reveals that the cell lines tend to have a higher fraction of genes mutated and higher median CNA compared to the tumours (Supplementary Fig. 1). In the set of 1,508 overlapping genes profiled for mutations by CCLE, CCLP and TCGA, the median number of mutated genes is 40 in CCLE kidney cell lines (minimum: 22, maximum: 92) and 26 in CCLP kidney cell lines (min 5, max 72) compared to 6 (min 0, max 27) in TCGA ccRCC tumours. With respect to copy number, the cell lines demonstrate a higher extent of CNAs than tumours (median fraction genome altered=0.49 in CCLE and 0.50 in CCLP cell lines, 0.13 in tumours). Only one cell line, SNU 349, is identified as a distinct outlier based on mutation counts (96/1651 genes mutated) and none are found to be outliers with respect to the extent of CNAs.

**Figure 3:**
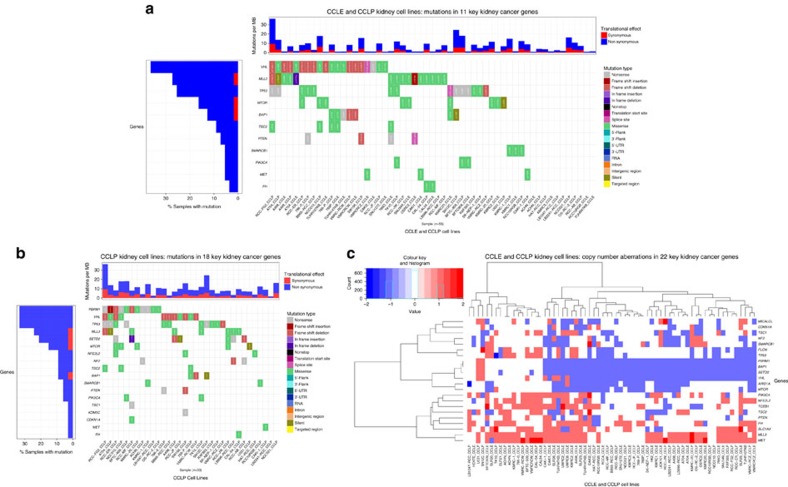
Mutations and CNAs in key kidney cancer genes in CCLP and CCLE cell lines. While CCLP provides mutation data for all 24 genes, CCLE only covers 16. Both provide CNA data for 22 genes. (**a**) Four CCLE cell lines (ACHN, KMRC3, RCC10RGB and TUHR4TKB) did not have any mutations in these key kidney cancer genes. None of the 22 CCLE cell lines with mutation data had mutations in *ARID1A, CDKN1A, FLCN1, NF2* or *TSC1*; while (**b**) none of the 33 CCLP cell lines with mutation data had mutations in *ARID1A, FLCN, MICALCL, SLC1A3, STAG2* or *TCEB1*. CAL-54 and 769-P have identical mutation data for these genes in CCLE and CCLP; while (**c**) CAL-54, ACHN and 786-O have perfect agreement of CNA data for the 22 genes included.

We next investigated the mutation data for 24 important genes (*TP53, VHL, PBRM1, SETD2, KDM5C, BAP1, NF2, PTEN, ARID1A, MICALCL, STAG2, SLC1A3, CDKN1A, MTOR, MET, SMARCB1, TCEB1, NFE2L2, PIK3CA, MLL3, FH, FLCN, TSC1, TSC2*) recurrently mutated in the three TCGA kidney cancer subtypes based on the three TCGA kidney cancer studies^17^,^22^,^24^. CCLP provides mutation data for all 24 genes (mutations reported in 18, Fig. 3a), whereas CCLE only includes 16 of these important genes (mutations reported in 11, Fig. 3b). While TCGA ccRCC tumours on average harbour only one mutation in these genes (with 22% tumours having no mutations in these genes, and 2, 3, 4 or 5 mutations found in 43%, 26%, 7.5%, 1.4% and 0.2% tumours, respectively), CCLP cell lines harbour 0–6 mutations, with a median of 2, and LB2241-RCC and NCC021 had no mutations in any of these key genes. In the 16 important kidney cancer genes covered by CCLE, the CCLE kidney cell lines had a range of 0–3 mutations and a median of 1 mutation, with *ACHN, KMRC1, KMRC3, SNU349, SNU1272, RCC10RGB* and *TUHR4TKB* showing no mutations in these key genes. None of the CCLP cell lines had a mutation in any of the genes *FLCN, ARID1A, MICALCL, SLC1A3, STAG2* or *TCEB1*; while none of the CCLE cell lines had a mutation in *FLCN, ARID1A, FLCN, NF2* or *TSC1*. Discrepancies between CCLE and CCLP for matching cell lines were observed, in line with a previous study^27^. Given that *VHL* is a notoriously challenging gene to sequence, we additionally culled the existing literature for evidence of *VHL* mutations in the various RCC cell lines (Supplementary Table 1). Interestingly, several cell lines that cluster with ccRCC and demonstrate the classical copy number characteristics do not harbour VHL mutations. Furthermore, mutations of *mTOR* pathway genes including *MTOR, TSC1, TSC2, PTEN* and *PIK3CA* are detected in nine (41%) of CCLE and 14 (42%) of the CCLP RCC cell lines.

### Comparison of alterations in key RCC genes in CCLE and CCLP

To address the discrepancies between CCLE and CCLP data, we investigated mutations and CNAs in 24 key kidney cancer genes (*TP53, VHL, PBRM1, SETD2, KDM5C, BAP1, NF2, PTEN, ARID1A, MICALCL, STAG2, SLC1A3, CDKN1A, MTOR, MET, SMARCB1, TCEB1, NFE2L2, PIK3CA, MLL3, FH, FLCN, TSC1* and *TSC2*) based on the three TCGA kidney cancer studies^17^,^22^,^24^ in detail (Fig. 3, also see Supplementary Table 3). While all 24 genes were present in the exome-wide mutation data provided by CCLP, the 1651 genes profiled by CCLE included 16 of these genes, so the comparison was restricted to 16 genes (*TP53, VHL, BAP1, NF2, PTEN, ARID1A, CDKN1A, MTOR, MET, SMARCB1, PIK3CA, MLL3, FH, FLCN, TSC1* and *TSC2*). Given a gene and a kidney cancer cell line common to CCLE and CCLP, we defined three tiers of mutations. Tier 1 consists of cases where both databases report identical mutations. Tier 2 consists of cases where both databases report mutations, but they are non-identical, and Tier 3 consists of cases where one database reports a mutation, while the other does not. Similarly, using 5-valued GISTIC scores for CNAs (−2: deep deletions, −1: shallow deletions, 0: no CNA, 1: low-level gain, 2: high-level amplification), we defined three tiers of CNAs. Tier 1 consists of cases where the databases agree on both nature and extent of CNA. Tier 2 consists of cases where the databases agree on the nature (gain/loss) but disagree on the extent, that is, one reports a high-level amplification while the other reports a low-level gain, or one reports a shallow loss while the other reports a deep deletion. Tier 3 consists of cases where one database reports a CNA, while the other reports either no CNA, or an alteration of in the ‘opposite' direction (gain versus loss).

This analysis revealed that 769P and CAL54 only have Tier 1 mutations in the key Kidney genes included in CCLE (see Fig. 3, also Supplementary Table 3), making them the ‘most reliable' in the sense of all their genomics alterations in key kidney cancer genes being confirmed by two independent sources.

The CNA analysis revealed that most disagreements are on the extent rather than nature of copy number aberrations between CCLE and CCLP in key kidney cancer genes. 786-O, ACHN, and CAL54 had perfect agreement on CNAs in key kidney cancer genes, while 769-P had only one disagreement (SMARCB1 is amplified in CCLE but diploid in CCLP).

Taken together, our analysis reveals CAL54 as the only cell line with perfect agreement on mutation and CNAs in key kidney cancer genes, with 769-P and a few other cell lines also showing a high degree of concordance. These cell lines might be thought of as the most trustworthy kidney cancer cell lines from the point of view of genomics-directed selection.

### Investigation of 3p loss as a hallmark of ccRCC

With respect to canonical copy number events (Fig. 2), we first investigated classical 3p loss^31^,^32^,^33^. To quantify 3p loss, we computed the fraction of chromosome 3p where the CNA data supported at least low-level copy number loss (using a log2 ratio). While this characteristic ccRCC genomic feature is observed in the majority of ccRCC cell lines, 3p loss is absent or significantly diminished in several of them, namely VMRCRCW, SLR20, SLR21, and BFTC909 (as well as the immortalized epithelial cell lines HK2 and HEKTE) in CCLE; and U031, KMRC-1, 786-0, VMRC-RCW, SN12C and BFTC-909 in CCLP (Supplementary Table 2). Of the cell lines lacking 3p loss, SLR21 and SLR20 in CCLE and SN12C and U031 in CCLP also lack other characteristic features of ccRCC such as chromosomal gains in five and eight or losses in chromosome 14, though SLR21 and U031 do show some gain in 8q.

### Alternative analysis of 3p loss using allele-specific data

Since CCLP provides allele-specific estimates of integral copy number (using PICNIC)^34^, we employed an alternative approach to estimate 3p loss, by computing the fraction of the chromosome arm for which the minor allele had a copy number of 0. This approach revealed that 786-O, KMRC-1 and VMRC-RCW had 3p loss, which was obfuscated by the major allele's amplification when using the log2 ratios of total copy number. When using the minor allele only, the cell lines with low/negligible 3p loss are SN12C, U031, SK-NEP-1, BFTC-909 and CAKI-1. All other CCLP kidney cell lines show a 3p loss≥80%. SK-NEP-1 and CAKI-1 have a minor allele copy number of 1 for most of 3p, and a total copy number of 2 for all or most of 3p, which indicated a loss relative to the average copy number of 2.62 and 3.23, respectively. Thus, combining the two approaches for estimating 3p loss in CCLP kidney cell lines, SN12C, U031 and BFTC-909 have negligible 3p loss according to both methods.

### Expression-based classification of ccRCC cell lines

**Figure 4:**
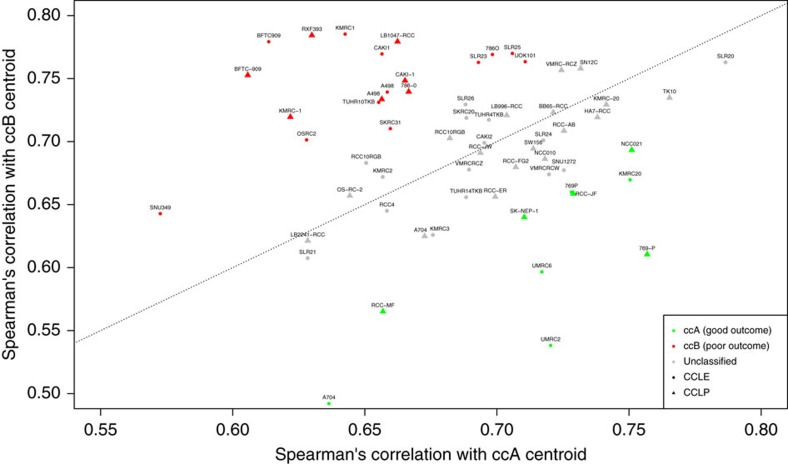
Predicted expression-based subtype of CCLE kidney cell lines. Most cell lines are not classified as either subtype with high confidence (grey)—of the remaining, more are classified as ccB (red) than as ccA (green).

We then analysed gene expression data to investigate whether the cell lines could be classified as the aforementioned prognostic expression-based subtypes ccA or ccB^25^. We found that of the 36 CCLE kidney cell lines, five (14%) classify as the more indolent ccA subtype, 13 (36%) classify as the more aggressive ccB subtype, while the remaining 18 (50%) are not assigned to either class, as their Spearman correlation with the centroids of the two classes differed by less than 0.05 (Fig. 4). Of the 10 most commonly cited ccRCC cell lines, three are classified as ccA (A-704, 769-P and UMRC2) and four are classified as ccB (CAKI-1, 786-O, A-498, OS-RC-2). The remaining 3 (RCC-4, CAKI-2, 769-P) are not predicted to be of either class. Similarly, of the 32 CCLP kidney cell lines with gene expression data, 8 are classified as ccB, 6 as ccA, and 18 are not classified as belonging to either class.

### Morphological correlations with particular cell lines

**Figure 5:**
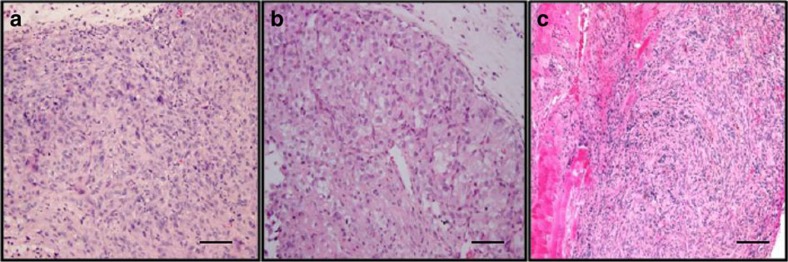
Cell Line Xenografts. Haematoxylin and eosin stain of tumour xenografts from the three most highly cited RCC cell lines (scale bar, 100 μm). (**a**) ACHN—xenografts show a poorly differentiated carcinoma with predominantly sarcomatoid differentiation. (**b**) A-498 xenografts consist of compact nests of tumour cells with clear cytoplasm, resembling the classical appearance of ccRCC. (**c**) 786-0 xenografts show predominantly sarcomatoid differentiation.

Owing to their genomic diversity and frequent use, we chose to perform xenografts on the three most highly cited RCC cell lines (ACHN, 786-0, A-498) in order to assess their morphologic features. In our cluster analysis, ACHN co-segregates with tumours displaying amplifications in chromosomes 7 and 17, furthering the notion that this appears to derive from papillary origins. In addition, it has been shown that Type 2 pRCC, which is the more aggressive form, frequently exhibits focal losses in chromosome 9p (ref. 35). ACHN shares this genotypic feature in our analysis, underscoring the aggressive nature of this cell line. Histologically, xenografts derived from ACHN cells appear to be a poorly differentiated carcinoma with predominantly sarcomatoid differentiation (Fig. 5a).

We then further investigated the two most highly cited cell lines that clustered with ccRCC but appear to have divergent genomic profiles. Our results indicate that 786-0 harbours more alterations than A-498 even though both cluster with ccRCC on a copy number level and harbour *VHL* mutations (Fig. 3). Consistent with these findings, xenograft tumours from A-498 consisted of compact nests of malignant epithelial cells with clear cytoplasm, a morphology resembling the classical appearance of ccRCC (Fig. 5b), whereas xenografts from 786-0 were characterized by poorly differentiated cells with sarcomatoid features (Fig. 5c).

## Discussion

Proper cell line selection is of paramount importance when investigating tumour biology. In a comparison of publicly available TCGA kidney tumours with CCLP and CCLE data for kidney cell lines, we have sub-classified commercially available RCC cell lines into their likely respective RCC sub-histologies; clear cell or papillary (none of the cell lines matched the chromophobe subtype). Previous studies have confirmed that certain RCC cell lines are truly derived from kidney tumours^5^,^36^ and others have categorized them into generic subgroups based on molecular signatures^37^. However, none of the previous analyses investigated which particular RCC subtype these cell lines originate from. After excluding the two CCLE cell lines derived from normal renal epithelium, we found that using CNA data, the vast majority of CCLE and CCLP cluster with ccRCC. However ACHN, among the most commonly referenced RCC cell line, clusters with a subset of papillary RCC. Although ACHN has been previously recognized to be of papillary origin based on chromosomal alteration patterns in 7 and 17 (refs 38, 39), several studies continue to utilize it as a standard model for RCC^40^,^41^,^42^. Similarly, U031 and CAL54, the other two cell lines that cluster with pRCC, also exhibit gains on chromosomes 7, 16 and 17, like ACHN. Another highly cited cell line, SN12C, is an outlier based on copy number aberrations. The remaining top ten most commonly cited cell lines all co-segregate with clear cell histology, though TK-10 displays an overall CNA landscape quite uncharacteristic of ccRCC (yet less dissimilar to ccRCC than to pRCC and chRCC). The other outlier based on CNAs—SLR20—has had only a few citations in the literature so far. Notably, a certain degree of heterogeneity exists within each of the clusters, which likely represents overlapping molecular features by particular tumours despite originating from unique subtypes in addition to the fact that some cell lines display a particularly high degree of genomic instability.

By comparing tumours which cluster with the cell lines based on CNAs with tumours that cluster away from the cell lines, we found that the tumours likely to be best represented by the cell lines carry hallmarks of aggressive disease, such as higher stage, higher grade, greater extent of CNAs, and more frequent mutations in genes such as *SETD2, BAP1* and *MTOR*, which have been associated with more aggressive disease and poorer outcomes.

In addition, we demonstrate that commonly used cell lines have higher fractional mutation rates and median CNAs than human tumours. These findings are consistent with a recent report by Beleut *et al*., which demonstrated that RCC cell lines had a higher mutational burden compared to primary tumours based on SNP profiling^37^. Only one of the cell lines we investigated, SNU349, is identified as a true outlier based upon fractional mutation rate, although its use has been limited in the scientific literature to date. There are several plausible explanations for the increased mutational rate among commercially available RCC cell lines. Primarily, several of these cell lines have been available for over two to three decades potentially undergoing genotypic and phenotypic alterations as a result of passaging and ongoing evolution. In addition, tumours tend to be infiltrated with stromal and immune cell components lacking detectable somatic mutations whereas cell lines are tumours in their purest form and thus will bear higher proportions of detectable genetic mutations. Finally, cell lines obtained from the CCLE and CCLP lack normal tissue for validation making it impossible to reliably filter out all germline events.

Further characterization of cell lines that cluster with ccRCC reveals considerable genomic variability with regards to copy number profile and mutations. Through integrative genomic analyses, we highlight the ccRCC cell lines that most closely resemble human tumours based on the presence or absence of characteristic features observed in this particular subtype. More specifically, we show that despite clustering with ccRCC, several of these cell lines lack *VHL* mutations. This finding may relate, in part, to selection pressures for growth of aggressive tumours that are subject to passaging effect over time. Other possibilities include known difficulties in sequencing the *VHL* gene as well as potential inactivation of the gene via promoter methylation. Moreover, our analysis reveals that genomic alterations with potential activating effect on the mTOR signalling pathway are detected in a significant portion of the ccRCC cell lines. Previous work from our group demonstrated that mTOR pathway activating mutations sensitize patients to rapalogs^43^,^44^, hence this new information may now be applied to *in vitro* work as well.

We address the discrepancy in genomic data from CCLE and CCLP via a detailed comparison of mutations in 15 key kidney cancer genes, and of CNAs in 18 key kidney cancer genes (Fig. 3, and Supplementary Table 3). By employing a tiered scheme to assess both nature and extent of disagreements, we discovered that CAL-54 and 769-P have perfect agreement on mutation data for these important genes in both databases, and 786-O, ACHN, and CAL-54 had perfect agreement on CNAs in key kidney cancer genes, while 769-P had only one disagreement (SMARCB1 is amplified in CCLE but diploid in CCLP). Thus, CAL-54 has the most reliable mutation and CNA data for key kidney cancer genes in terms of validation via two independent sources, while 769-P is a close second.

A growing interest in the prognostic ability of the mRNA-based genetic signature ccA/ccB^25^,^26^ led to our additional analysis of the ccRCC cell lines. In a recent systematic assessment of 28 ccRCC prognostic biomarkers, ccB was the only one that added additional independent prognostic value to routine clinical evaluation^45^, therefore this information may be of particular relevance both experimentally and clinically. In our study, we find that while some cell lines have a stronger correlation with one of the subtypes and more are classified as ccB than ccA, most cell lines have a comparable correlation with each class, meaning they cannot be reliably classified as either. The lack of a strong correlation with either class might reflect the widespread differences in the transcriptomes of cell lines and tumours^2^,^3^. The fact that more cell lines classify as the ccB subtype might be not be surprising given that there is a known bias in cell line collections towards aggressive tumours. However, when selecting the appropriate cell line, one may consider tailoring their particular experiment according to tumour behaviour in addition to tumour subtype.

Finally, we assessed the morphological architecture of the three most highly cited RCC cell lines (ACHN, 786-0, A-498) in order to further investigate how the genomic landscape of these cell lines translates histologically. Despite ACHN clustering with pRCC based on CNA data as well as previous reports suggesting similar original histology^20^,^38^,^46^, sarcomatoid differentiation rather than a papillary architecture is observed in our murine model. However, according to the 2004 WHO classification of renal tumours, it is known that sarcomatoid differentiation can be found in any of the recognized subtypes of RCC and typically reflects a high-grade nature of the corresponding tumour^47^. With respect to the two most highly cited cell lines that cluster with ccRCC, we demonstrate that their unique genomic profiles lend to distinctive morphologic features; 786-0 appearing poorly differentiated with sarcomatoid features while A-498 displays epithelial cells with clear cytoplasm, a morphology more akin to the classical appearance of ccRCC. These observations highlight the fact that while both cell lines likely derive from ccRCC, 786-0 appears to have undergone significant de-differentiation both genomically and morphologically.

We acknowledge the limitations of this study, including the lack of complete mutational and expression data for every cell line despite utilizing several publically available resources and exploring the available literature. In addition, a relatively restricted number of genes were sequenced for CCLE and multiple sequencing platforms were applied in the various analyses used in this study. Furthermore, several discrepancies were found between CCLE and CCLP, especially in mutation data, as previously reported by others^27^, which we addressed by stratifying the overlapping cell lines by consistency between CCLE and CCLP, yielding a set of high-confidence cell lines with reliable data on alterations in key kidney cancer genes. While the analysis of allele-specific CNA data from CCLP yielded different results on LOH in chromosome 3p for some cell lines than those based on the analysis of log2 ratios (abundances) in CCLP and CCLE, we regard the additional insights generated by combining data from CCLE and CCLP as a strength of this study, as it allowed us to characterize a greater number of renal cell lines across these two major resources in greater detail than focusing on either resource exclusively would have.

In summary, we utilize publically available genomic data from TCGA, CCLP and CCLE to compare the molecular profiles of human RCC tumours to those of commercially available cell lines. We show that the vast majority of cell lines resemble ccRCC tumours, but the highly cited ACHN cell line resembles pRCC. We also show that tumours that are most likely to be well represented by cell lines tend to carry hallmarks of aggressive disease, and conversely, most cell lines resemble the expression-based ccRCC subtype associated with more aggressive disease. This study may therefore serve as a guide for future investigators as to the suitability of particular RCC cell lines for *in vitro* examination.

## Methods

### Data acquisition

Mutation, CNA and gene expression data for CCLE kidney cancer cell lines was obtained from the CCLE website^8^, and for CCLP cell lines from the COSMIC Cell Lines Project website^48^ via SFTP. Mutation data for KIRC, and CNA data for KIRC, KIRP and KICH TCGA data sets were obtained from the Broad Institute Genomic Data Analysis Centre (GDAC) website^49^. Training data for gene expression-based subtype classification—expression levels (of 6386 genes) and class labels for 480 KIRC tumours—was kindly provided by Rose Brannon and Kimryn Rathmell.

### Mutation analysis

To compare mutation counts, we used the mutation data available from CCLE and TCGA, which excluded various kinds of putative neutral and common variants. We further excluded mutations from intronic, untranslated region, flanking and intergenic regions, as well as silent and RNA mutations. To compare mutations across the same set of genes, we only used TCGA data for the same 1,651 genes for which CCLE provides mutation data. CCLP and CCLE mutation data was compared using the 1543 genes present in both data sets. For CCLE, we used the file listed as ‘preferred data set' by CCLE, that is: CCLE_hybrid_capture1650_hg19_NoCommonSNPs_NoNeutralVariants_CDS_2012.05.07.maf. This dataset filters out variants that are any of the following: common polymorphisms, have an allelic fraction of <10%, are located outside the CDS for all transcripts, or are putative neutral variants based on low conservation in vertebrates. CCLP only provided one dataset, which had been filtered for likely germline variants by comparison with ∼8,000 normal data sets (from 1,000 Genomes, ESP6500, DBSNP and an in-house dataset of 350 normals, as described in ref. 50 and a confidence filter requiring read depth ≥15 and mutant allele burden≥15%. These filters are stricter than those employed by CCLE and thus likely to filter out more false positives—for the comparison of mutation counts (Supplementary Fig. 1) and similarity using mutation data (Fig. 1), we applied the read depth and allelic fraction requirements of CCLP to the CCLE data, and also filtered out variants of unknown effect from the CCLP data (using their data on ‘Mutation description' in the above mentioned file). For the analysis of mutation in key kidney cancer genes, we chose not to further filter the CCLE data due to the risk of inadvertently removing mutations in key cancer genes^27^. Mutati1on heatmaps (oncoprints) were created using the oncoprinter tool of the cBio cancer genomics portal^51^.

### Copy number analysis

For CCLE, we used the file ‘CCLE_copynumber_2012-09-29.seg' from the CCLE website, and for CCLP, the file ‘cell_lines_copy_number.csv' from the CCLP website. Since CCLP provided segmented data with estimated integral copy numbers rather than log2 ratios (as CCLE and TCGA did), we converted the CCLP data to log2 ratios by computing the average total copy number per sample, dividing the integral copy number of each segment by the average total copy number, and taking the logarithm to the base 2. Correlating this with CCLE data revealed a high inter-data set similarity among matched cell lines, confirming that the conversion was meaningful.

Fraction Genome Altered (FGA) was calculated as follows for a given log2 (sample intensity/reference intensity) value *CN*, a threshold *T* and a length *L(i)* of segment *i*:


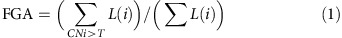


In other words, FGA is the ratio of the sum of the lengths of all segments with signal above the threshold, to the sum of all segment lengths. A log2 (sample intensity/reference intensity) threshold of 0.2 (for amplification, −0.2 for deletion) was used for both the TCGA tumour samples as well as the CCLE cell lines. The fraction of chromosome 3p lost was similarly calculated using a threshold of −0.2.

For clustering CNA data, we used the gene-wise copy number data for KIRC, KIRP, KICH and CCLE and CCLP kidney cell lines, and (1—Spearman's correlation) as the distance. Hierarchical clustering was employed with average inter-cluster distance based agglomeration for combining sub-clusters.

### Comparing KIRC tumours and cell line clustering

To compare the TCGA KIRC tumours which grouped with or away from the majority of kidney cancer cell lines from CCLE and CCLP (Fig. 2a), we cut the dendrogram (tree) at a height of 0.9, yielding 6 clusters—C1, a KICH-dominated subtree of 74 members (55 out of 66 KICH tumours, 17 KIRC and 2 KIRP tumours); C2, a five-member subtree of four KIRP tumours and one KIRC tumour; C3; a KIRC-dominated subtree of 167 tumours (158 KIRC, six KIRP, two KICH) and a solitary cell line, KMRC-3; C4, a 422 member subtree consisting of the vast majority of cell lines (57 out of 65) and a majority of KIRC tumours (315 out of 504), along with 41 KIRP and 9 KICH tumours; C5, a KIRP-dominated subtree (105 out of 158 KIRP tumours, 13 KIRC tumours) with five cell lines (ACHN and CAL-54 from both CCLE and CCLP, and U031 from CCLP); and finally C6, an ‘outlier' subtree of the cell lines SN12C and SLR20. We compared the KIRC tumours in subtree C4 with the rest of KIRC tumours with respect to stage, grade, extent of CNA and frequency of mutations in 22 key kidney cancer genes (mutation data was available for 415 KIRC tumours, of which 267 clustered with cell lines (were in subtree C4), and 148 did not.

### Comparison of mutations and CNAs in key kidney cancer genes

To resolve the discrepancies between CCLP and CCLE data, we compared the mutation and CNA data between kidney cancer cell lines in common for 16 out of 24 key kidney cancer genes (since CCLE only includes these 16 genes among the 1651 genes it screened for mutations). For mutations in a given gene and cell line, we defined three ‘tiers' of mutations, depending on the extent of disagreement between the two databases. Tier 1 consists of cases with identical mutations in both CCLE and CCLP. Tier 2 comprises cases with non-identical mutations in the same gene—while these are discrepancies, they are often close to each other and could potentially be the same mutation, with the discrepancy a result of alignment and other technical issues. Tier 3 consists of cases where a mutation is reported in one database, but not in the other.

Similarly, for CNAs, we defined three tiers using GISTIC scores (+2—high-level amplification, +1—gain, 0—no alteration, −1: shallow loss, −2: deep deletion) for a given gene and CNA. Tier 1 comprises cases where CCLP and CCLE agree on the nature and amplitude/extent of the CNA. Tier 2 consists of cases where CCLP and CCLE agree on the nature but disagree on the amplitude/extent of the CNA, that is, one database reports a high-level amplification but the other reports a low-level gain, or one reports a shallow loss while the other reports a deep deletion. Tier 3: consists of cases where a CNA is reported in one database, but not the other.

### Gene expression analysis

For CCLE, we used the file ‘CCLE_Expression_Entrez_2012-09-29.gct' from the CCLE website, and for CCLP, we obtained the data from ArrayExpress^52^ (accession code E-MTAB-3610)^9^. For classification into the expression-based subtypes ccA or ccB, we used the PAMR classifier^53^, which uses shrunken centroids in order to emphasize the most discriminative genes. Training data of 6,386 genes and 480 samples was filtered to retain only the 5,980 genes which were present in the CCLE and CCLP data and only the 412 tumours which were classified as only ccA or ccB (244 and 168, respectively). Since we were using three different data sources, the combat function of the sva package^54^,^55^ was used for batch-correction before training the classifier (and for comparing CCLE and CCLP gene expression data). The best classification performance on the training data with 10-fold cross-validation was achieved using a threshold of 3.7 and 780 genes, for which the classification error was 3.7% for ccA and 3.6% for ccB. Therefore, we computed the Spearman's correlation coefficient of each cell line with the centroid of each class using these 780 genes—if the correlation of a cell line with a given subtype was at least 0.05 than the correlation with the other subtype, it was classified as the respective subtype; otherwise it was not classified as either subtype.

All programming was done in Perl and R^56^, and statistical calculations were done using R. The R packages, dendextend^57^, gplots and corrplot were used to plot coloured dendrograms, heatmaps and correlation/similarity matrices, and the Bioconductor package GenVisR^58^ was used to plot mutation waterfall plots.

The number of Pubmed Central articles mentioning one of the CCLE kidney cancer cell lines was determined with the Pubmed Central search builder using several punctuation alternatives for the cell line names (Supplementary Table 1).

### Xenografting

All mouse experiments were performed using an approved protocol under Memorial Sloan-Kettering Cancer Center's Institutional Animal Care and Use Committee. For subcutaneous growth, 4 million cells were mixed 2:1 with Matrigel (BD Biosciences) and injected into NSG mice (The Jackson Laboratory). When the tumour reached 300–400 mm^3^ in volume, mice were euthanized and tumour was collected for histological analysis. For haematoxylin and eosin staining, tissue samples were fixed in 10% formalin and embedded in paraffin. Sections of 5 μm thickness were prepared. haematoxylin and eosin staining was performed as per standard protocol. Each slide was individually reviewed by an experienced genitourinary pathologist (Y.B.C.).

### Data availability

Databases used in this study are the Cancer Cell Line Encyclopedia^8^, the COSMIC Cell Lines Project^48^, ArrayExpress^52^ with accession code E-MTAB-3610, and the Broad TCGA GDAC center^49^. Processed data from these databases are available from the authors upon request.

## Additional information

**How to cite this article:** Sinha, R. *et al*. Analysis of renal cancer cell lines from two major resources enables genomics-guided cell line selection. *Nat. Commun.* **8,** 15165 doi: 10.1038/ncomms15165 (2017).

**Publisher's note:** Springer Nature remains neutral with regard to jurisdictional claims in published maps and institutional affiliations.

## Supplementary Material

Supplementary Information Supplementary Figure and Supplementary Tables

## Figures and Tables

**Table 1:** Number of kidney cell lines and the data types available in CCLE and CCLP.

**Source**	**No. kidney cell lines**	**Mutation data**	**Copy number data**	**Gene expression data**	**No. Cell lines with all three data types**
CCLE	36	22 (1,651 Genes)	33	36	22
CCLP	33	33 (Whole exome)	32	32	31

CCLE, Broad-Novartis Cancer Cell Line Encyclopedia; CCLP, COSMIC Cell Lines Project.

## References

[b1] Ertel A., Verghese A., Byers S. W., Ochs M. & Tozeren A. Pathway-specific differences between tumor cell lines and normal and tumor tissue cells. Mol. Cancer 5, 55 (2006). 17081305 10.1186/1476-4598-5-55 PMC1635729

[b2] Stein W. D., Litman T., Fojo T. & Bates S. E. A Serial Analysis of Gene Expression (SAGE) database analysis of chemosensitivity: comparing solid tumors with cell lines and comparing solid tumors from different tissue origins. Cancer Res. 64, 2805–2816 (2004). 15087397 10.1158/0008-5472.can-03-3383

[b3] Gillet J. P. . Redefining the relevance of established cancer cell lines to the study of mechanisms of clinical anti-cancer drug resistance. Proc. Natl Acad. Sci. USA 108, 18708–18713 (2011). 22068913 10.1073/pnas.1111840108 PMC3219108

[b4] Sandberg R. & Ernberg I. Assessment of tumor characteristic gene expression in cell lines using a tissue similarity index (TSI). Proc. Natl Acad. Sci. USA 102, 2052–2057 (2005). 15671165 10.1073/pnas.0408105102 PMC548538

[b5] Wang H. . Comparative analysis and integrative classification of NCI60 cell lines and primary tumors using gene expression profiling data. BMC Genomics 7, 166 (2006). 16817967 10.1186/1471-2164-7-166 PMC1525183

[b6] *The Cancer Genome Atlas*. Available at: https://cancergenome.nih.gov/.

[b7] Barretina J. . The Cancer Cell Line Encyclopedia enables predictive modelling of anticancer drug sensitivity. Nature 483, 603–607 (2012). 22460905 10.1038/nature11003 PMC3320027

[b8] *Cancer Cell Line Encyclopedia.* Available at: http://www.broadinstitute.org/ccle/home.

[b9] Iorio F. . A landscape of pharmacogenomic interactions in cancer. *Cell* **166**, 740–754 (2016). 10.1016/j.cell.2016.06.017 PMC4967469 27397505

[b10] Garnett M. J. . Systematic identification of genomic markers of drug sensitivity in cancer cells. Nature 483, 570–575 (2012). 22460902 10.1038/nature11005 PMC3349233

[b11] Domcke S., Sinha R. & Levine D. A. Evaluating cell lines as tumour models by comparison of genomic profiles. Nat. Commun. 4, 2126 (2013). 23839242 10.1038/ncomms3126 PMC3715866

[b12] Li H. . Genomic analysis of head and neck squamous cell carcinoma cell lines and human tumors: a rational approach to preclinical model selection. Mol. Cancer Res. 12, 571–582 (2014). 24425785 10.1158/1541-7786.MCR-13-0396 PMC3989421

[b13] Ferlay J. . Estimates of worldwide burden of cancer in 2008: GLOBOCAN 2008. Int. J. Cancer 127, 2893–2917 (2010). 21351269 10.1002/ijc.25516

[b14] Hsieh J. J. . Renal cell carcinoma. Nat Rev Dis Primers 3, 17009 (2017). 28276433 10.1038/nrdp.2017.9 PMC5936048

[b15] Sato Y. . Integrated molecular analysis of clear-cell renal cell carcinoma. Nat. Genet. 45, 860–867 (2013). 23797736 10.1038/ng.2699

[b16] Guo G. . Frequent mutations of genes encoding ubiquitin-mediated proteolysis pathway components in clear cell renal cell carcinoma. Nat. Genet. 44, 17–19 (2012). 10.1038/ng.1014 22138691

[b17] consortium, T. Comprehensive molecular characterization of clear cell renal cell carcinoma. Nature 499, 43–49 (2013). 23792563 10.1038/nature12222 PMC3771322

[b18] Shen C. . Genetic and functional studies implicate HIF1alpha as a 14q kidney cancer suppressor gene. Cancer Discov. 1, 222–235 (2011). 22037472 10.1158/2159-8290.CD-11-0098 PMC3202343

[b19] Delahunt B. & Eble J. N. Papillary renal cell carcinoma: a clinicopathologic and immunohistochemical study of 105 tumors. Mod. Pathol. 10, 537–544 (1997). 9195569

[b20] Schmidt L. . Germline and somatic mutations in the tyrosine kinase domain of the MET proto-oncogene in papillary renal carcinomas. Nat. Genet. 16, 68–73 (1997). 9140397 10.1038/ng0597-68

[b21] Lubensky I. A. . Hereditary and sporadic papillary renal carcinomas with c-met mutations share a distinct morphological phenotype. Am. J. Pathol. 155, 517–526 (1999). 10433944 10.1016/S0002-9440(10)65147-4 PMC1866853

[b22] Linehan W. M. . Comprehensive molecular characterization of papillary renal-cell carcinoma. N. Engl. J. Med. 374, 135–145 (2015). 26536169 10.1056/NEJMoa1505917 PMC4775252

[b23] Storkel S. . Classification of renal cell carcinoma: Workgroup No. 1. Union Internationale Contre le Cancer (UICC) and the American Joint Committee on Cancer (AJCC). Cancer 80, 987–989 (1997). 9307203 10.1002/(sici)1097-0142(19970901)80:5<987::aid-cncr24>3.0.co;2-r

[b24] Davis C. F. . The somatic genomic landscape of chromophobe renal cell carcinoma. Cancer Cell 26, 319–330 (2014). 25155756 10.1016/j.ccr.2014.07.014 PMC4160352

[b25] Brannon A. R. . Molecular stratification of clear cell renal cell carcinoma by consensus clustering reveals distinct subtypes and survival patterns. Genes Cancer 1, 152–163 (2010). 20871783 10.1177/1947601909359929 PMC2943630

[b26] Brannon A. R. . Meta-analysis of clear cell renal cell carcinoma gene expression defines a variant subgroup and identifies gender influences on tumor biology. Eur. Urol. 61, 258–268 (2012). 22030119 10.1016/j.eururo.2011.10.007 PMC3244546

[b27] Hudson A. M. . Discrepancies in cancer genomic sequencing highlight opportunities for driver mutation discovery. Cancer Res. 74, 6390–6396 (2014). 25256751 10.1158/0008-5472.CAN-14-1020 PMC4247168

[b28] Smith M. A. . SK-NEP-1 and Rh1 are Ewing family tumor lines. Pediatr. Blood Cancer 50, 703–706 (2008). 17154184 10.1002/pbc.21099

[b29] Naito S., von Eschenbach A. C., Giavazzi R. & Fidler I. J. Growth and metastasis of tumor cells isolated from a human renal cell carcinoma implanted into different organs of nude mice. Cancer Res. 46, 4109–4115 (1986). 3731078

[b30] Bear A. . Characterization of two human cell lines (TK-10, TK-164) of renal cell cancer. Cancer Res. 47, 3856–3862 (1987). 3594443

[b31] Kovacs G. . Consistent chromosome 3p deletion and loss of heterozygosity in renal cell carcinoma. Proc. Natl Acad. Sci. USA 85, 1571–1575 (1988). 2894030 10.1073/pnas.85.5.1571 PMC279815

[b32] Jonasch E. . State of the science: an update on renal cell carcinoma. Mol. Cancer Res. 10, 859–880 (2012). 22638109 10.1158/1541-7786.MCR-12-0117 PMC3399969

[b33] Kroeger N. . Deletions of chromosomes 3p and 14q molecularly subclassify clear cell renal cell carcinoma. Cancer 119, 1547–1554 (2013). 23335244 10.1002/cncr.27947

[b34] Greenman C. D. . PICNIC: an algorithm to predict absolute allelic copy number variation with microarray cancer data. Biostatistics. 11, 164–175 (2010). 19837654 10.1093/biostatistics/kxp045 PMC2800165

[b35] Klatte T. . Cytogenetic and molecular tumor profiling for type 1 and type 2 papillary renal cell carcinoma. Clin. Cancer Res. 15, 1162–1169 (2009). 19228721 10.1158/1078-0432.CCR-08-1229

[b36] Dietlein F. & Eschner W. Inferring primary tumor sites from mutation spectra: a meta-analysis of histology-specific aberrations in cancer-derived cell lines. Hum. Mol. Genet. 23, 1527–1537 (2014). 24163242 10.1093/hmg/ddt539

[b37] Beleut M. . Integrative genome-wide expression profiling identifies three distinct molecular subgroups of renal cell carcinoma with different patient outcome. BMC Cancer 12, 310 (2012). 22824167 10.1186/1471-2407-12-310 PMC3488567

[b38] Borden E. C., Hogan T. F. & Voelkel J. G. Comparative antiproliferative activity *in vitro* of natural interferons alpha and beta for diploid and transformed human cells. Cancer Res. 42, 4948–4953 (1982). 7139598

[b39] Anglard P. . Molecular and cellular characterization of human renal cell carcinoma cell lines. Cancer Res. 52, 348–356 (1992). 1345811

[b40] Yao J. . Decreased expression of a novel lncRNA CADM1-AS1 is associated with poor prognosis in patients with clear cell renal cell carcinomas. Int. J. Clin. Exp. Pathol. 7, 2758–2767 (2014). 25031695 PMC4097296

[b41] Dong X. . hZIP1 that is down-regulated in clear cell renal cell carcinoma is negatively associated with the malignant potential of the tumor. Urol. Oncol. 32, 885–892 (2014). 24878177 10.1016/j.urolonc.2014.02.021

[b42] Ma X. . Dicer is down-regulated in clear cell renal cell carcinoma and in vitro Dicer knockdown enhances malignant phenotype transformation. Urol. Oncol. 32, 46.e49–17 (2014). 10.1016/j.urolonc.2013.06.011 24094887

[b43] Voss M. H. . Tumor genetic analyses of patients with metastatic renal cell carcinoma and extended benefit from mTOR inhibitor therapy. Clin. Cancer Res. 20, 1955–1964 (2014). 24622468 10.1158/1078-0432.CCR-13-2345 PMC4140619

[b44] Xu J. . Mechanistically distinct cancer-associated mTOR activation clusters predict sensitivity to rapamycin. J. Clin. Invest. 126, 3526–3540 (2016). 27482884 10.1172/JCI86120 PMC5004947

[b45] Gulati S. . Systematic evaluation of the prognostic impact and intratumour heterogeneity of clear cell renal cell carcinoma biomarkers. Eur. Urol. 66, 936–948 (2014). 25047176 10.1016/j.eururo.2014.06.053 PMC4410302

[b46] Kovacs G., Fuzesi L., Emanual A. & Kung H. F. Cytogenetics of papillary renal cell tumors. Genes Chromosomes Cancer 3, 249–255 (1991). 1958590 10.1002/gcc.2870030403

[b47] Lopez-Beltran A., Scarpelli M., Montironi R. & Kirkali Z. 2004 WHO classification of the renal tumors of the adults. Eur. Urol. 49, 798–805 (2006). 16442207 10.1016/j.eururo.2005.11.035

[b48] *COSMIC Cell Lines Project*. Available at: http://cancer.sanger.ac.uk/cancergenome/projects/cell_lines/.

[b49] *The Broad Institute Genomic Data Analysis Centre (GDAC) Website*. Available at: http://gdac.broadinstitute.org/.

[b50] *Cancer Genome Annotation in the COSMIC Cell Lines Project*. Available at: https://grch37-cancer.sanger.ac.uk/cell_lines/analyses.

[b51] Cerami E. . The cBio cancer genomics portal: an open platform for exploring multidimensional cancer genomics data. Cancer Discov. 2, 401–404 (2012). 22588877 10.1158/2159-8290.CD-12-0095 PMC3956037

[b52] Kolesnikov N. . ArrayExpress update--simplifying data submissions. Nucleic Acids Res. 43, D1113–D1116 (2015). 25361974 10.1093/nar/gku1057 PMC4383899

[b53] Tibshirani R., Hastie T., Narasimhan B. & Chu G. Diagnosis of multiple cancer types by shrunken centroids of gene expression. Proc. Natl Acad. Sci. USA 99, 6567–6572 (2002). 12011421 10.1073/pnas.082099299 PMC124443

[b54] Johnson W. E., Li C. & Rabinovic A. Adjusting batch effects in microarray expression data using empirical Bayes methods. Biostatistics. 8, 118–127 (2007). 16632515 10.1093/biostatistics/kxj037

[b55] Leek J. T., Johnson W. E., Parker H. S., Jaffe A. E. & Storey J. D. The sva package for removing batch effects and other unwanted variation in high-throughput experiments. Bioinformatics. 28, 882–883 (2012). 22257669 10.1093/bioinformatics/bts034 PMC3307112

[b56] R Core Team. R: A language and environment for statistical computing. R Foundation for Statistical Computing, Vienna, Austria (2016). https://www.R-project.org/.

[b57] Galili T. dendextend: an R package for visualizing, adjusting and comparing trees of hierarchical clustering. Bioinformatics. 31, 3718–3720 (2015). 26209431 10.1093/bioinformatics/btv428 PMC4817050

[b58] Skidmore Z. L. . GenVisR: Genomic Visualizations in R. Bioinformatics. 32, 3012–3014 (2016). 27288499 10.1093/bioinformatics/btw325 PMC5039916

