# Supplementary material for: Analysis of renal cancer cell lines from two major resources enables genomics-guided cell line selection
Source: Nat Commun. 2017 May 10;8:15165. doi: 10.1038/ncomms15165 (PMC5436135; doi:10.1038/ncomms15165)
Supplement: Supplementary Information — Supplementary Figure and Supplementary Tables [file ncomms15165-s1.pdf]

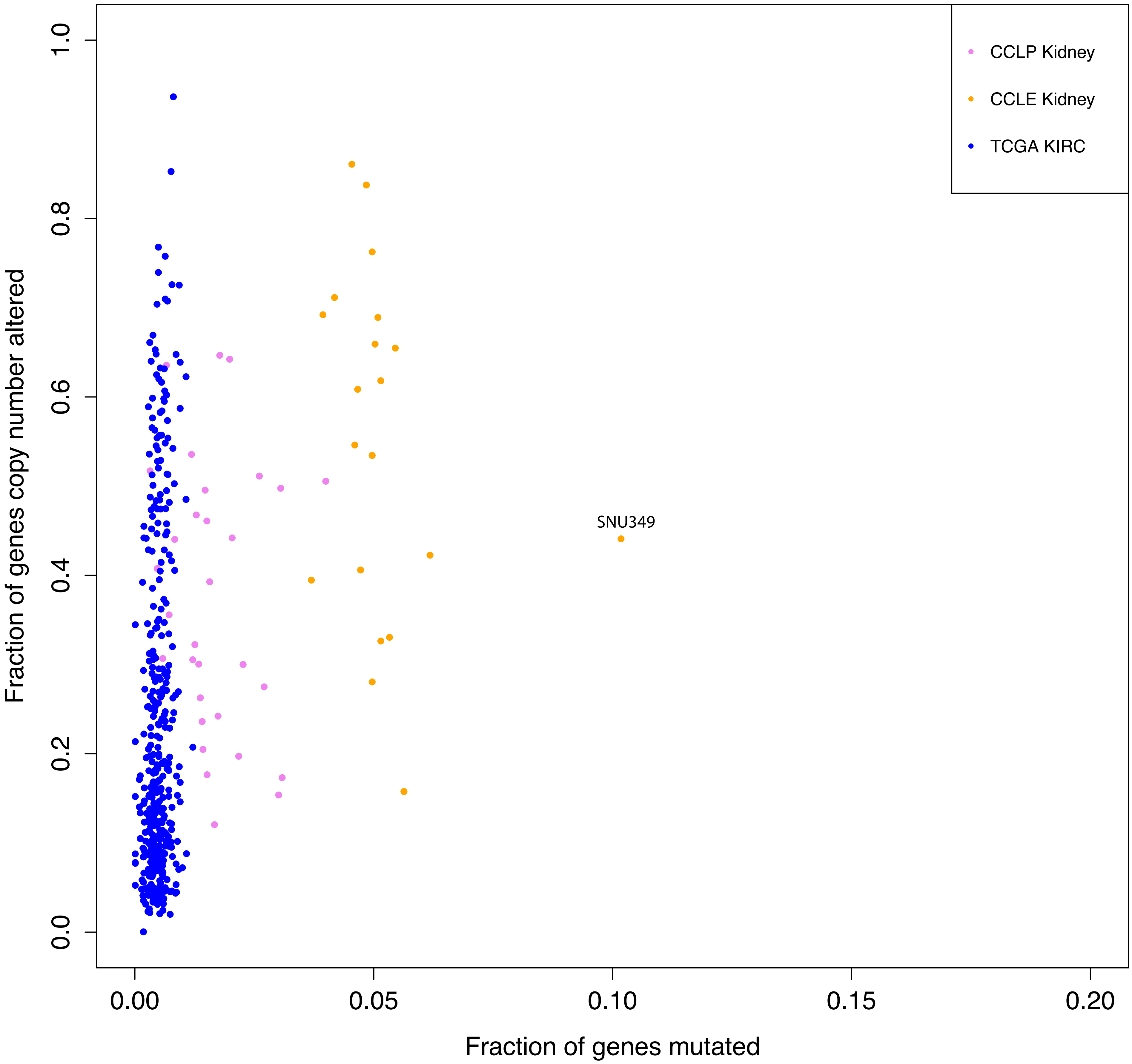

Supplementary Figure 1. Mutational burden and copy number alterations in RCC cell lines and tumors. Comparison of mutational burden and copy number alterations in TCGA clear cell renal tumors to CCLP and CCLE kidney cancer cell lines – SNU349 is a clear outlier based on mutation count.

Supplementary Table 1. Number of citations, VHL mutation status and subtype of CCLE and CCLP kidney cell lines (inferred by copy number clustering with TCGA kidney cancer datasets).

Cell Line	Cell line Alias	Pubmed Central citations	Subtype Clustering	VHL
HK2*	HK-2	538	normal kidney	
CCLE				NA
COSMIC				NA
Other Source				wt (Raval, Mol Cell Bio, 2005)
786O	786-0	401	ccRCC	
CCLE				wt
COSMIC				mutated
Other Source				mutated (Maxwell,Nature , 1999)
A498	A-498	381	ccRCC	
CCLE				mutated
COSMIC				mutated
Other Source				mutated (Shinojima, Carcinogenesis, 2007)
ACHN		364	pRCC	
CCLE				wt
COSMIC				wt
Other Source				wt (Razorenova, PNAS, 2011)
CAKI1	CAKI-1	344	ccRCC	
CCLE				wt
COSMIC				NA
Other Source				wt (Raval, Mol Cell Bio, 2005)
RCC4	RCC-4	226	ccRCC	
CCLE				NA
COSMIC				NA
Other Source				mutated (Shen, Cancer Discovery, 2011)
A704	A-704, A.704	187	ccRCC	
CCLE				mutated
COSMIC				mutated
Other Source				mutated (Whaley, Am J Hum Genet, 1994)
SN12C	SN-12C	162	Outlier	
CCLE				NA
COSMIC				wt
Other Source				wt (Turcotte, Cancer Cell, 2008)
CAKI2	CAKI-2	158	ccRCC	
CCLE				mutated
COSMIC				NA
Other Source				mutated (Whaley, Am J Hum Genet,1994)
769P	769-P	102	ccRCC	
CCLE				mutated
COSMIC				mutated
Other Source				mutated (Whaley, Am J Hum Genet,1994)
SK-NEP-1	SKNEP-1, SKNEP1	76	ccRCC	
CCLE				NA
COSMIC				wt
Other Source				no information
TK10	TK-10	72	ccRCC	
CCLE				NA
COSMIC				wt
Other Source				wt (LaGory, Cell Reports, 2015)
RXF393	RXF-393	33	ccRCC	
CCLE				NA
COSMIC				wt
Other Source				wt (Kuzmin, Oncogene 1999)
UMRC2	UMRC-2	29	ccRCC	
CCLE				NA
COSMIC				NA
Other Source				mutated (Isaacs, J. Biol. Chem, 2002)
OSRC2	OS-RC-2	20	ccRCC	
CCLE				wt
COSMIC				mutated
Other Source				no information
UMRC6	UMRC-6	16	ccRCC	
CCLE				NA
COSMIC				NA
Other Source				mutated (Chen, Cancer Research, 1995)
UOK101	UOK-101	11	ccRCC	
CCLE				NA
COSMIC				NA
Other Source				mutated (Gnarra, Nature Genetics, 1994)
TUHR10TKB	TUHR-10TKB	9	ccRCC	
CCLE				mutated
COSMIC				NA
Other Source				no information
VMRCRCW	VMRC-RCW	7	ccRCC	
CCLE				mutated
COSMIC				NA
Other Source				no information
U031		7	pRCC	
CCLE				NA
COSMIC				wt
Other Source				wt (Escobar, Cancer Res, 2010)
SW156	SW-156	6	ccRCC	
CCLE				NA
COSMIC				mutated
Other Source				mutated (Beroukhim, Cancer Res, 2009)
CAL54	CAL-54	6	pRCC	
CCLE				wt
COSMIC				wt
Other Source				no information
TUHR4TKB	TUHR-4TKB	5	ccRCC	
CCLE				wt
COSMIC				NA
Other Source				no information
LB996-RCC	LB996RCC	4	ccRCC	
CCLE				NA
COSMIC				wt
Other Source				no information
SLR20	SLR-20	4	Outlier	
CCLE				NA
COSMIC				NA
Other Source				wt (Shen, Cancer Discovery, 2011)
RCC10RGB	RCC-10RGB	4	ccRCC	
CCLE				wt
COSMIC				wt
Other Source				no information
BFTC909	BFTC-909	3	ccRCC	
CCLE				wt
COSMIC				wt
Other Source				no information
HA7-RCC	HA7RCC	3	ccRCC	
CCLE				NA
COSMIC				wt
Other Source				no information
LB1047-RCC	LB1047RCC	3	ccRCC	
CCLE				NA
COSMIC				wt
Other Source				no information
KMRC3	KMRC-3	2	ccRCC	
CCLE				wt
COSMIC				NA
Other Source				mutated (Tanaka, J Biol Chem, 2012)
BB65-RCC	BB65RCC	2	ccRCC	
CCLE				NA
COSMIC				mutated
Other Source				no information
TUHR14TKB	TUHR-14TKB	2	ccRCC	
CCLE				mutated
COSMIC				NA
Other Source				no information
SLR21	SLR-21	2	ccRCC	
CCLE				NA
COSMIC				NA
Other Source				wt (Shen, Cancer Discovery, 2011)
HEKTE*		1	normal kidney	
CCLE				NA
COSMIC				NA
Other Source				no information
KMRC1	KMRC-1	1	ccRCC	
CCLE				wt
COSMIC				NA
Other Source				mutated (Matsuura, BMC Cancer , 2011)
KMRC20	KMRC-20	1	ccRCC	
CCLE				wt
COSMIC				NA
Other Source				no information
KMRC2	KMRC-2	1	ccRCC	
CCLE				wt
COSMIC				NA
Other Source				no information
SNU1272	SNU-1272	1	ccRCC	
CCLE				mutated
COSMIC				NA
Other Source				mutated (Ku, Cancer Res Treat, 2005)
SLR26	SLR-26	1	ccRCC	
CCLE				NA
COSMIC				NA
Other Source				mutated (Shen, Cancer Discovery, 2011)
SLR25	SLR-25	1	ccRCC	
CCLE				NA
COSMIC				NA
Other Source				no information
SLR23	SLR-23	1	ccRCC	
CCLE				NA
COSMIC				NA
Other Source				no information
VMRCRCZ	VMRC-RCZ	1	ccRCC	
CCLE				mutated
COSMIC				mutated
Other Source				no information
SNU349	SNU-349	1	ccRCC	
CCLE				wt
COSMIC				NA
Other Source				mutated (Ku, Cancer Res Treat, 2005)
RCC-ER	RCCER	1	ccRCC	
CCLE				NA
COSMIC				mutated
Other Source				no information
RCC-FG2	RCCFG2	1	ccRCC	
CCLE				NA
COSMIC				mutated
Other Source				no information
RCC-JW	RCCJW	1	ccRCC	
CCLE				NA
COSMIC				wt
Other Source				no information
LB2241-RCC	LB2241RCC	0	ccRCC	
CCLE				NA
COSMIC				wt
Other Source				no information
NCC010		0	ccRCC	
CCLE				NA
COSMIC				mutated
Other Source				no information
NCC021		0	ccRCC	
CCLE				NA
COSMIC				wt
Other Source				no information
RCC-AB	RCCAB	0	ccRCC	
CCLE				NA
COSMIC				wt
Other Source				no information
RCC-JF	RCCJF	0	ccRCC	
CCLE				NA
COSMIC				mutated
Other Source				no information
RCC-MF	RCCMF	0	ccRCC	
CCLE				NA
COSMIC				wt
Other Source				no information

Legend

* Denotes cell line derived

DNC=Does not classify with

NA= Data not available

WT= Wild-type

Supplementary Table 2a. Chromosome 3p loss estimates for CCLE and CCLP cell lines (based on log2 ratios)

Cell Line	Chromosome 3p loss (based on log2 ratio ≤ -0.2)
769-P_CCLP	0.92
786-0_CCLP	0.00
A498_CCLP	1.00
A704_CCLP	0.94
ACHN_CCLP	1.00
BB65-RCC_CCLP	0.99
BFTC-909_CCLP	0.03
CAKI-1_CCLP	0.66
CAL-54_CCLP	1.00
HA7-RCC_CCLP	0.80
KMRC-1_CCLP	0.33
KMRC-20_CCLP	0.99
LB1047-RCC_CCLP	1.00
LB2241-RCC_CCLP	0.91
LB996-RCC_CCLP	1.00
NCC010_CCLP	1.00
NCC021_CCLP	1.00
OS-RC-2_CCLP	1.00
RCC-AB_CCLP	1.00
RCC-ER_CCLP	1.00
RCC-FG2_CCLP	1.00
RCC-JF_CCLP	0.80
RCC-JW_CCLP	1.00
RCC-MF_CCLP	1.00
RCC10RGB_CCLP	0.97
RXF393_CCLP	0.99
SK-NEP-1_CCLP	1.00
SN12C_CCLP	0.01
TK10_CCLP	1.00
U031_CCLP	0.00
VMRC-RCW_CCLP	0.01
VMRC-RCZ_CCLP	1.00
769P_CCLE	0.78
786O_CCLE	0.97
A498_CCLE	1.00
A704_CCLE	0.92
ACHN_CCLE	0.56
BFTC909_CCLE	0.07
CAKI1_CCLE	1.00
CAKI2_CCLE	1.00
CAL54_CCLE	0.70

HEKTE_CCLE	0.00
HK2_CCLE	0.00
KMRC1_CCLE	1.00
KMRC20_CCLE	1.00
KMRC2_CCLE	0.82
KMRC3_CCLE	1.00
OSRC2_CCLE	1.00
RCC10RGB_CCLE	0.97
RCC4_CCLE	1.00
SLR20_CCLE	0.00
SLR21_CCLE	0.00
SLR23_CCLE	1.00
SLR25_CCLE	0.67
SLR26_CCLE	0.82
SNU1272_CCLE	1.00
SNU349_CCLE	1.00
TUHR10TKB_CCLE	1.00
TUHR14TKB_CCLE	0.63
TUHR4TKB_CCLE	0.58
UMRC2_CCLE	0.67
UMRC6_CCLE	1.00
UOK101_CCLE	0.97
VMRCRCW_CCLE	0.00
VMRCRCZ_CCLE	1.00

Supplementary Table 2b. Chromosome 3p loss estimates for CCLP cell lines(based on minor allele copy number = 0).

CCLP Cell Line	Chromosome 3p loss (minor allele copy number = 0)
769-P	0.92
786-0	1.00
A498	1.00
A704	0.94
ACHN	1.00
BB65-RCC	1.00
BFTC-909	0.08
CAKI-1	0.09
CAL-54	1.00
HA7-RCC	0.81
KMRC-1	1.00
KMRC-20	0.99
LB1047-RCC	1.00
LB2241-RCC	0.92
LB996-RCC	1.00
NCC010	1.00
NCC021	1.00
OS-RC-2	1.00
RCC-AB	1.00
RCC-ER	1.00
RCC-FG2	1.00
RCC-JF	0.82
RCC-JW	1.00
RCC-MF	1.00
RCC10RGB	0.98
RXF393	1.00
SK-NEP-1	0.05
SN12C	0.01
TK10	1.00
U031	0.04
VMRC-RCW	0.99
VMRC-RCZ	1.00

Supplementary Table 3. Mutations in key kidney cancer genes in kidney cancer cell lines in CCLE and CCLP.
Tier 1 (Green): Identical mutations in given gene,
Tier 2 (Blue): Non-identical mutations reported by CCLE and CCLP,
Tier 3 (Red): Discrepancy in mutational status – only one of the databases reports a mutation.

Cell Line	ARID1A	BAP1	FH	FLCN	MET	MLL3	MTOR	PIK3CA	PTEN	SMARCB1	TP53	TSC2	VHL	KDM5C	CDKN1A	NF2	NFE2L2	PBRM1	SETD2	TSC1
769.P_CCLP	-	p.Y33D	-	-	-	-	-	-	-	-	-	p.I606V	p.I180N	-	-	-	-	-	-	-
769P_CCLE	NA	p.Y33D	-	-	-	-	-	-	-	-	-	p.I606V	p.I180N	NA	-	-	NA	NA	NA	-
786.0_CCLP	-	-	-	-	-	-	-	-	p.Q149*	-	p.?_p.P278A	-	p.G104fs*55	-	-	-	-	-	-	-
786O_CCLE	NA	-	-	-	-	p.A3902G	-	-	p.Q149*	-	p.R248W	p.D1126V	-	NA	-	-	NA	NA	NA	-
A498_CCLP	-	-	-	-	-	p.G2986D	-	-	-	-	-	-	p.G144fs*14	-	-	-	-	-	p.V2033fs*9	-
A498_CCLE	NA	-	-	-	-	p.G2986D	-	-	-	-			p.V142fs	NA	-	-	NA	NA	NA	-
A704_CCLP	-	-	-	-	-	p.P3633P	-	-	-	-	R_p.Y126D_p	-	p.I180N	-	-	-	-	p.E572fs*16	-	-
A704_CCLE	NA	-	-		-	p.H475del	-	-	-	-	-	-	p.I180N	NA	-	-	NA	NA	NA	-
ACHN_CCLP	-	-	-	-	-	-	-	-	-	-	-	-	-	-	-	p.R57*	-	p.R1052*	-	-
ACHN_CCLE	NA	-	-	-	-	-	-	-	-	-	-	-	-	NA	-	-	NA	NA	NA	-
BB65.RCC_CCLP	-	-	-	-	-	-	-	-	-	-	p.R248W	-	p.P192fs*10	-	-	-	-	-	-	-
BFTC.909_CCLP	-	-	-	-	-	-	-	p.E545K	-	-	E224*_p.S24	-	-	-	-	-	-	p.G1177S	-	-
BFTC909_CCLE	NA	-	-	-	-	-	-	p.E545K	-	-	S241C_p.E22	-	-	NA	-	-	NA	NA	NA	-
CAKI.1_CCLP	-	-	-	-	p.V1238I	-	-	-	-	-	-	-	-	-	-	-	-	-	p.?	-
CAKI1_CCLE	NA	-	-	-	p.V1238I	p.G892E	-	-	-	-	-	-	-	NA	-	-	NA	NA	NA	-
CAKI2_CCLE	NA	-	-	-	-	-	-	-	-	-	-	-	p.R177*	NA	-	-	NA	NA	NA	-
CAL.54_CCLP	-	-	p.I120V	-	-	p.C3460F	-	-	-	-	-	-	-	-	-	-	-	-	-	-
CAL54_CCLE	NA	-	p.I120V	-	-	p.C3460F	-	-	-	-		-	-	NA	-	-	NA	NA	NA	-
HA7.RCC_CCLP	-	-	-	-	-	-	-	-	-	-	-	-	-	-	-	-	p.D27A	-	-	-
KMRC1_CCLE	NA	-	-	-	-	-	-	-	-	p.P156S	-	-	-	NA	-	-	NA	NA	NA	-
KMRC.1_CCLP	-	-	-	-	-	-	-	-	p.?	p.P165S	-	-	-	-	-	-	-	p.W1183*	-	-
KMRC20_CCLE	NA	p.E31K	-	-	-	-	p.W1304R	-	-	-	p.E224_splice	-	-	NA	-	-	NA	NA	NA	-
KMRC.20_CCLP	-	-	-	-	-	-	p.W1304R	-	-	-	p.?	-	p.S168fs*5	-	-	-	-	p.N453Y	-	-
KMRC2_CCLE	NA	-	-	-	-	-	p.Y1974H	-	-	-	-	-	-	NA	-	-	NA	NA	NA	-
KMRC3_CCLE	NA	-	-	-	-	-	-	-	-	-	-	-	-	NA	-	-	NA	NA	NA	-
LB1047.RCC_CCLP	-	-	-	-	-	-	-	-	-	-	-	-	-	-	-	p.?	-	p.R1245G	-	-
LB2241.RCC_CCLP	-	-	-	-	-	-	-	-	-	-	-	-	-	-	-	p.?	-	-	-	-
LB996.RCC_CCLP	-	-	-	-	-	p.S3080P	-	-	-	-	-	-	-	-	-	p.A105fs*20	-	-	p.G1141*	-
NCC010_CCLP	-	-	-	-	-	-	p.L2209V	-	-	-	-	p.D559V	p.V130F	-	-	-	-	p.K1269*	-	-
NCC021_CCLP	-	-	-	-	-	-	-	-	-	-	p.?	-	-	-	-	-	-	-	-	-
OS.RC.2_CCLP	-	-	-	-	-	-	-	-	p.?	-	-	-	-	-	-	-	-	p.I233T	-	-
OSRC2_CCLE	NA	-	-	-	-	p.Y816fs	-	-	.N212_splice	-	-	-	-	NA	-	-	NA	NA	NA	-
RCC.AB_CCLP	-	-	-	-	-	-	-	-	-	-	-	-	-	-	-	p.K294*	-	-	p.R1629fs*15	-
RCC.ER_CCLP	-	-	-	-	-	-	p.P770A	-	-	-	p.R248W	-	p.D143fs*16	-	-	-	p.G31A	p.D251fs*31	p.L152*	-
RCC.FG2_CCLP	-	-	-	-	-	fs*73_p.P15	-	-	-	-	R213Q_p.R21	.E75K_p.E75	fs*11_p.F14	-	-	-	-	Q477*_p.Q47	-	-
RCC.JF_CCLP	-	-	-	-	-	-	-	-	-	-	-	-	p.H115Y	-	-	-	-	-	p.A879A	-
RCC.JW_CCLP	-	-	-	-	-	p.P2326Q	p.L2138F	-	-	-	-	-	-	-	-	-	-	-	p.G756V	-
RCC.MF_CCLP	-	-	-	-	-	p.G2474E	-	-	-	-	-	-	-	-	p.P79H	-	-	p.N528fs*41	257_H1259de	p.Y761*
RCC10RGB_CCLP	-	p.?_p.?	-	-	-	-	-	-	-	p.R377H	-	-	-	-	-	-	-	-	-	-
RCC10RGB_CCLE	NA	-	-	-	-	-	-	-	-	-	-	-	-	NA	-	-	NA	NA	NA	-
RXF393_CCLP	-	-	-	-	-	-	-	-	-	-	p.R175H	-	-	-	-	-	-	-	-	-
SK.NEP.1_CCLP	-	-	-	-	-	-	-	-	-	-	p.G245S	-		-	-	-	-	-	-	-
SN12C_CCLP	-	p.F168F	-	-	-	-	-	-	-	-	p.E336*	-	-	-	-	p.?	-	-	-	-
SNU1272_CCLE	NA	-	-	-	-	-	-	-	-	-	-	-	p.H115Q	NA	-	-	NA	NA	NA	-
SNU349_CCLE	NA	-	-	-	-	p.T107A	p.E1799K	-	-	-	-	-	-	NA	-	-	NA	NA	NA	-
SW156_CCLP	-	-	-	-	-	-	-	-	p.T321fs*23	-	-	-	p.L153fs*6	p.E465*	-	-	Q489K_p.D7	-	-	-
TK10_CCLP	-	-	-	-	-	388P_p.D48	-	p.L719V	-	-	p.L264R	-	-	-	-	-	-	-	-	-
TUHR10TKB_CCLE	NA	-	-	-	-	-	p.V2006L	-	-	-	-	-	p.E186fs	NA	-	-	NA	NA	NA	-
TUHR14TKB_CCLE	NA	p.S287*	-	-	-	-	-	-	-	-		-	p.L153P	NA	-	-	NA	NA	NA	-
TUHR4TKB_CCLE	NA	-	-	-	-	-	-	-	-	-	-	-	-	NA	-	-	NA	NA	NA	-
U031_CCLP	-	-	-	-	-	-	p.L2209L	-	-	-	-	-	-	-	-	-	-	-	-	-
VMRC.RCW_CCLP	-	p.K580fs*37	-	-	-	-	-	-	-	-	p.?	-	p.R161fs*12	-	-	-	-	-	-	-
VMRCRCW_CCLE	NA	p.K580fs	-	-	-	-	-	-	-	-	-	-	p.E160fs	NA	-	-	NA	NA	NA	-
VMRCRCZ_CCLE	NA	-	-	-	p.E168D	-	-	-	-	-	-		p.V155_splice	NA	-	-	NA	NA	NA	-
VMRC.RCZ_CCLP	-	-	-	-	-	-	-	-	-	-	p.D48fs*74	-	p.?	-	-	-	-	-	-	-
